# Pregnant beef cow’s nutrition and its effects on postnatal weight and carcass quality of their progeny

**DOI:** 10.1371/journal.pone.0237941

**Published:** 2020-08-27

**Authors:** Daniele Zago, Maria Eugênia Andrighetto Canozzi, Júlio Otávio Jardim Barcellos

**Affiliations:** 1 Department of Animal Science, Federal University of Rio Grande do Sul, Porto Alegre, RS, Brazil; 2 Instituto Nacional de Investigación Agropecuaria (INIA), Programa Producción de Carne y Lana, Estación Experimental INIA La Estanzuela, Colonia, Uruguay; Universidade Federal de Viçosa, BRAZIL

## Abstract

A systematic review (SR) and meta-analysis (MA) were performed to evaluate the effects of different energy levels (metabolizable energy, ME) and crude protein (CP), supplied to pregnant cows, on weight of their progenies at 60 (BW60), 100 (BW100), 180 (BW180) and 205 (BW205) days of age, average daily gain (ADG), and weight, age, loin eye area (LEA), marbling and fat thickness (FT) at slaughter. The SR was performed on two electronic databases. The MA for random effects was performed for each response variable separately. The BW60 was reduced (P<0.001; *I*^*2*^ = 78.9%) when cows consumed CP and ME above the required levels during the third trimester of pregnancy (3TRI). The BW205 was lower (P<0.001; *I*^*2*^ = 92.6%) when cows consumed ME above the recommended levels in the second trimester of pregnancy (2TRI) and 3TRI. Conversely, the ADG was higher when cows consumed CP (P = 0.032; *I*^*2*^ = 96.1%) and ME (P<0.001; *I*^*2*^ = 96.1%) above the required levels. The steers whose mothers consumed CP and ME above the required levels during the 3TRI were slaughtered 5.5 days earlier (P = 0.015; *I*^*2*^ = 98.5%) compared to other steers. The marbling was higher (P<0.001; *I*^*2*^ = 91.7%) in calves born to mothers consuming CP and ME above the recommended levels, regardless of the gestation phase. The FT was higher (P<0.001; *I*^*2*^ = 0%) in the offspring of cows that consumed CP and ME above the required levels during the 3TRI. Thus, CP and ME intake, at levels higher than those recommended by the NRC, by pregnant cows in the 3TRI reduces the progeny weight up to 205 days of age. However, this is advantageous during the finishing phase, as it reduces slaughter age and increases the ADG and carcass quality by improving marbling and FT.

## Introduction

Maternal nutritional status is one of the factors involved in fetal programming, through nutrient partitioning during pre and postnatal growth and development of fetal tissues [1]. Mammals are born with a predetermined number of muscle and fat cells, and after birth, these cells only increase in size by a hypertrophic process [2].

In cattle, beef quality is dependent on postnatal nutrition and prenatal development, i.e., development of muscles and fat tissues [3–5]. Furthermore, the nutritional management may be more effective during the prenatal rather than the postnatal period of an animal's life [2].

It is believed that the fetus adapts its development when the mother is faced with nutritional challenges during pregnancy. These adaptations can occur through the production of hormones [3, 6, 7], or through changes in the expression of genes responsible for body composition [4, 8–10] both of which affect live weight and carcass characteristics, which are measures of high commercial interest [9]. These changes might be due to overfeeding [4, 7] or maternal subnutrition [11–13].

Overfeeding of pregnant cows can increase fat thickness, finishing degree, and carcass yield on slaughter of their progeny [7], i.e., it improves adipogenesis without compromising fetal myogenesis [4]. However, energy oversupply can be detrimental to fetal development, leading to low birth weight [14]. Conversely, maternal malnutrition has been reported to reduce the fetal loin eye area and calf weight at 105 days of age [12].

These data show the importance of fetal programming in cattle. An understanding of mechanisms that generate these responses allows the development of strategies to increase muscle and fat growth in the cattle offspring [2]. However, the results of previously published work are conflicting, and a systematic review (SR) and meta-analysis (MA) can potentially clarify these concepts. Hence, our objective was to evaluate the different levels of energy (metabolizable energy, ME) and crude protein (CP) consumption in beef cows during pregnancy, and their relationship with weight at various postnatal stages, slaughter weight, and carcass characteristics of their progenies, through an SR-MA.

## Materials and methods

### Research question and protocol

The present study is theoretical and does not require an evaluation by the ethics committee.

The current study aims to identify the effects of different dietary metabolizable energy (ME) and crude protein (CP) levels fed to pregnant beef cows on postnatal weight and carcass quality of their progeny. The literature search strategy was defined based on the main concepts in terms the following of PICO: population, intervention, comparator and outcome. For population terms were used: ‘cow calf’ or ‘beef cattle’ or ‘beef heifer’ or ‘beef dams’; for intervention: nutrition or energy or supplemen* or protein or feed* or aliment*; and for outcome: ‘foetal programming’ or ‘foetal growth’ or ‘birth weight’ or ‘compensatory growth’ or ‘weaning weight’ or ‘quality of muscle fibre’ or adipogenesis or myogenesis.

The population studied was pregnant cows and heifers. The interventions of interest were dietary energy and protein levels fed to pregnant cows and heifers. Similar groups of animals subjected to the same treatment with or without intervention were considered as comparative groups. This study continues the data already published [14] about weight of fetuses and calves, however, in this study the outcomes of interest were progeny body weight at 60 (BW60), 100 (BW100), 180 (BW180) and 205 (BW205) days of age; average daily gain (ADG) in the finishing period to slaughter; age, weight, loin eye area (LEA), marbling and fat thickness (FT) at slaughter. To be relevant to our SR, the studies should include at least one of the outcomes of interest. A search protocol was developed, and each screening tool was adapted from forms applied in earlier studies [14–16]. The protocol was tested before being implemented.

### Search methods for the identification of studies

Two electronic databases were used: Scopus (Elsevier, 1960–2016) and Web of Science (Clarivate Analytics, 1945–2016). The research was conducted in October 2016 and updated in August 2019. The search verification included a manual search in the references of two relevant literature reviews on the topic [17, 18]. All references have been exported to software EndNote Web® (Clarivate Analytics, Jersey, England) for organizing and manually removing duplicate references.

### Study selection criteria and relevance screening

Four reviewers were trained for the relevance screening steps using 30 abstracts. In this stage, potentially relevant studies were identified. The abstracts were assessed independently by two reviewers by reading the title, abstract and keywords. No language or year of publication restrictions were applied. When the response of all reviewers was ‘no’ to one or more questions, the reference was excluded. The conflicts were solved by agreement; if no agreement was reached, another reviewer was consulted. Randomized and non-randomized studies were included. The Microsoft Excel® software was used during all relevant screening stages.

#### Methodological assessment and data extraction process

The data extraction form was developed based on previous models. Manuscripts reporting more than one trial were duplicated and the data extracted as separate studies to obtain as much detail as possible. Information extracted from each study was divided into general information (study population, intervention, parameters evaluated and outcome data) and manuscript-related information (authors, publication year and original language).

#### Considerations for data collection and manipulation

For each outcome, a database including the mean, the standard error of the mean or other available dispersion measure, the probability value and the number of animals evaluated in each group (control and treatment) was built.

In order to compare dietary nutrient supply, the levels of ME (Mcal/kg) and CP (g/kg) fed to the cows were separately calculated as a percentage (%) of their requirements [19]. When the studies did not report ME and CP dietary levels, these were calculated based on the amount of each ingredient that composed the diets, multiplied by their ME and CP content (%) as mentioned in NRC (1996) [19]. For each comparison, differences in ME and CP levels between the treatment and control groups were calculated. The control group always received nutrient supply equal to the demands and the treatment group levels above or below. When studies reported the probability value, the standard deviation was estimated using the t-statistic, assuming that the data presented a normal distribution, according to the following equation [15, 20]: *S*_*p*_ = (*x*_2 −_*x*_1_) _/_ t(*αdf*E)√(1/*n*_*2*_) + (1/*n*_*1*_), where *x*_*2*_*−x*_*1*_ represents the difference between the means, t*(αfdE)* is the percentile of the reference distribution and *n* is the sample size of each group.

#### Quality assessment

The Cochrane Collaboration Risk Bias Tool [21] was used to assess the risk of publication bias in individual studies included in this meta-analysis (MA). However, interpretation of the risk of bias due to blind use of the outcome assessors was considered low for all outcomes, since it was measured using a scale or other objective measurement equipment.

### Meta-analysis

Studies included in this MA reported enough quantitative data to estimate the mean difference (MD) between the control and the treatment groups and 95% confidence interval (95% CI). For the postnatal weight results, the values obtained referred to period observed in each study (60, 100, 180 and 205 postnatal days). For ADG, age, weight, marbling, FT and LEA at slaughter (pre slaughter) it was considered the measurement periods of each study.

Since each study had different experimental designs, between-study heterogeneity was assumed and estimated using the DerSimonian and Laird method [22]. All statistical analyses were performed using software Stata V 14.0 (StataCorp., Texas, USA).

Each outcome was evaluated separately as a group, and a pooled effect on MD and 95% CI (forest plot) was generated. Cochran’s *Q* test (χ^*2*^ test of heterogeneity) and *I*^*2*^ (percentage of total variation across studies due to heterogeneity rather than chance) were calculated based on dietary ME and CP levels and on the outcome of interest. The magnitude of *I*^*2*^ was interpreted in the order of 25 (low), 50 (moderate) and, 75% (high) [23].

### Publication bias

Publication bias was evaluated graphically (funnel plot) and statistically (Begg’s correlation and Egger’s linear regression tests) for each outcome of interest. If there was any trend in publication bias (*P*< 0.10), the ‘trim and fill’ method was applied to estimate the extent of bias [24]. This method indicates the number of studies that should be included in the analysis to achieve symmetry in the weight distribution graph.

### Meta-regression

Univariate MA was performed to explore the sources of data heterogeneity, applying the method-of-moments approach [25].The following variables were explored: randomization (yes or no); cluster control (not applicable, systematic, convenience or deliberate, randomized, not reported); confounders identified and controlled (no, yes or not applicable); year of publication; continent (North America, South America, Central America, Asia, Oceania, Europe or Africa); dam and sire groups (*Bos indicus*, *Bos taurus* (British breeds) purebreds, *B*. *indicus* × *B*. *Taurus* (British breeds) crossbreds, *B*. *indicus* × *B*. *taurus* (Continental breeds) crossbreds, *B*. *Taurus* (British × Continental) crossbreds, *B*. *indicus* × *B*. *Taurus* (British and Continental crossbreds); evaluation period (days), sample size; parity (primiparous or multiparous); gestation period (first (1TRI), second (2TRI) or third (3TRI) trimester); production system (grazing or feedlot); and body condition score (1–9 scale [26]) (S1 Table).

### Cumulative MA and sensitivity analysis

Cumulative MA are often performed to update the overall treatment effect each time a new study is published. It allows to identify the time when the treatment effect was significant relative to the control. Sensitivity analyses were conducted to verify whether determined studies influenced the measure of effect (MD), by manually removing one study at a time and evaluating whether the MD varied ±30% before including the next study.

## Results

### Study selection

The search identified 2470 citations, of which 443 full-texts were read in their entirety to assess their eligibility. Of these, 389 texts were excluded after methodological validation and data extraction (S1 Fig), and eight of the remaining manuscripts did not report sufficient data for performing the quantitative analyses (S1 and S2 Tables). Finally, 25 publications on postnatal body weight, age, weight, ADG, LEA, marbling, and FT at slaughter were included in this SR-MA (Table 1).

**Table 1:** Descriptive summary of each relevant study included in this meta-analysis and meta-regression [35].

Comparative group	Test diets (control/treatment)	Number of studies/Sample size	Outcome parameter	Reference	Country
Energy levels	H/H and C	2/128	BW205	[27]	USA
	H and C/ H and C	2/101	BW180	[28]	USA
	H/ Fo	1/198	BW205, age and weight at slaughter, LEA	[29]	Canada
	C/ Si and C	1/160	BW60, BW100 and BW205	[30]	USA
	H, Fo and C/ H, Fo and C	3/342	BW180	[31]	USA
	H and C/Fo	1/20	BW205, ADG, LEA, FT, age, weight and marbling at slaughter	[32]	USA
	H and C/H and C	1/233	BW205	[33]	USA
	Fo, H and C/ Fo, H and C	1/11	FT, LEA and marbling at slaughter	[34]	USA
	H, Si and C/ H, Si and C	1/30	BW205	[35]	USA
	Fo and C/ Fo and C	1/514	BW205	[36]	USA
	H and C/ H and C	1/13	BW60	[37]	USA
	H/ H and C	1/270	BW100 and BW180	[6]	USA
	Fo and C/ Fo and C	7/658	BW205	[38]	USA
	H/ H and Si	1/276	BW180 and BW205	[39]	USA
	Fo/ Fo and C	1/177	BW60, BW100, ADG, FT, LEA, age, weight and marbling at slaughter	[40]	USA
	Fo/ CR and C	1/24	BW180 and BW205	[41]	USA
	H, S and C/ H, S and C	1/68	LEA	[42]	Australia
	H/ H and C	1/114	ADG, FT, LEA and marbling at slaughter	[43]	USA
	Fo and C/ Fo	1/362	BW180 and BW205	[44]	USA
Protein levels	H/ C and CR	1/163	BW100, FT, LEA, age, weight and marbling at slaughter	[45]	USA
	H/H	1/71	BW205	[46]	Canada
Energy and protein levels	H and C/ H and C	1/36	ADG, age and weight at slaughter	[47]	USA
	Fo /Fo and C	1/20	ADG, FT, LEA, age, weight and marbling at slaughter	[8]	USA
	Fo, H and C/ Fo, H and C	1/96	BW180	[48]	USA
	H and C/ H and C	1/118	BW100 and BW205	[49]	USA

H = hay.

C = concentrate.

Fo = forrage.

Si = silage.

S = barley straw.

CR = corn residue.

BW60 = weight at 60 days of life.

BW100 = weight at 100 days of life.

BW180 = weight at 180 days of life.

BW205 = weight at 205 days of life.

ADG = average daily gain.

FT = fat thickness.

LEA = loin eye area.

Twenty-five publications were included in this SR-MA, representing 35 studies, 140 comparisons, and 8,208 animals (Table 2).

**Table 2:** Descriptive characteristics of 25 publications reporting 35 studies included in the meta-analysis.

Variable	Categories	Number of publications (studies)
Study design	Control studies	25 (35)
Treatment (type of nutrient)	Energy level	15 (25)
	Protein level	5 (5)
	Energy and protein level	5 (5)
Year of publication	1962–2000	7 (15)
	2000–2016	18 (20)
Parity	Primiparous	20 (22)
	Multiparous	2 (3)
	Primiparous and multiparous	3 (10)
Gestation period in which the study was conducted	First trimester	2 (3)
	Second trimester	6 (8)
	Third trimester	10 (10)
	First and second trimester	3 (4)
	Second and third trimester	2 (8)
	All pregnancy	2 (2)
Cows suckling or not calf of previous gestation	Suckling	2 (2)
	No suckling	21 (31)
	Suckling and no sukling	1 (1)
	No data	1 (1)
Production system	Extensive system	15 (23)
	Intensive system	10 (12)
Experiment period	1 to 90 days	9 (16)
	90 to 180 days	12 (15)
	180 to 280 days	4 (4)
Genetic description of dams	*Bos taurus*—British	16 (25)
	Cross *Bos indicus* x *Bos taurus*—British	1 (1)
	Cross *Bos indicus*, *Bos taurus* british, *Bos taurus* continental	1 (1)
	*Bos taurus* continental x *Bos taurus* british	6 (7)
	*Bos taurus* british and cross *Bos taurus* continental x *Bos taurus* british	1 (1)
Genetical description of sires	*Bos taurus*—British	7 (8)
	*Bos taurus*—Continental	3 (10)
	Cross *Bos indicus* x *Bos taurus* british	1 (1)
	Cross *Bos indicus*, *Bos taurus* british, *Bos taurus* continental	1 (1)
	*Bos taurus* continental and *Bos taurus* british	1 (1)
	Not reported	12 (14)
Continent	South America	1 (1)
	North America	24 (34)
Sample size	n<50	8 (8)
	n = 51 a 100	3 (3)
	n>100	14 (24)

The number of studies considered for each outcome was: BW60 (n = 3 studies), BW100 (n = 5 studies), BW180 (n = 10 studies), BW205 (n = 20 studies), age (n = 6 studies), weight at slaughter (n = 6 studies), ADG at slaughter (n = 5 studies), LEA (n = 8 studies), marbling (n = 6 studies) and FT (n = 6 studies). Fifteen studies evaluating the ME consumption of pregnant cows, six evaluating the CP consumption, and six evaluating both ME and CP were also included in this study.

### Risk of bias

Unlike objective measurements (postnatal weight, age, ADG, LEA, FT, and slaughter weight), analysis of marbling was a subjective measure, as it was performed by an evaluator (visual interpretation) using a numeric scale (Marbling Score—USDA). The studies included in this SR-MA did not mention the blindness of the evaluator; hence, the risk of bias was considered “unclear”. We found that several studies had failed to detail the publication bias information (S1 and S2 Tables and Table 3).

**Table 3:** Methodological quality assessment of risk of bias (classified as low, unclear and high) of the 35 studies included in the MA of the effect of cow nutrition on body weight at 60, 100, 180 and 205 days of life, age, weight and average daily gain at slaughter, loin eye area, marbling and fat thickness at slaughter.

Reference	Sequence generation	Allocation concealment	Selective reporting	Outcome measurement	Blinding of outcome assessment	Incomplete outcome data
[27]	High	Unclear	Unclear	BW205	Low	Low
[28]	High	Unclear	Unclear	BW180	Low	Low
[29]	High	Unclear	Unclear	BW205, LEA, age and weight at slaughter	Low	Low
[30]	High	Unclear	Unclear	BW60, BW100 and BW205	Low	Low
[31]	High	Unclear	Unclear	BW180	Low	Low
[41]	High	Unclear	Unclear	BW180 and BW205	Low	Low
[32]	High	Unclear	Unclear	BW205, ADG, LEA, FT, age, weight and marbling at slaughter	Unclear	Low
[33]	High	Unclear	Unclear	BW205	Low	Low
[47]	High	Unclear	Unclear	ADG, age and weight at slaughter	Low	Low
[42]	High	Unclear	Unclear	LEA	Low	Low
[34]	High	Unclear	Unclear	FT, LEA and marbling at slaughter	Unclear	Low
[8]	High	Unclear	Unclear	ADG, FT, LEA, age, weight and marbling at slaughter	Unclear	Low
[35]	High	Unclear	Unclear	BW205	Low	Low
[36]	High	Unclear	Unclear	BW205	Low	Low
[37]	High	Unclear	Unclear	BW60	Low	Low
[6]	High	Unclear	Unclear	BW100 and BW180	Low	Low
[38]	High	Unclear	Unclear	BW205	Low	Low
[39]	High	Low	Unclear	BW180 and BW205	Low	Low
[43]	High	Low	Unclear	ADG, FT, LEA and marbling at slaughter	Unclear	Low
[44]	High	Low	Unclear	BW180 and BW205	Low	Low
[40]	High	Unclear	Unclear	BW60, BW100, ADG, FT, LEA, age, weight and marbling at slaughter	Unclear	Low
[45]	High	Unclear	Unclear	BW100, FT, LEA, age, weight and marbling at slaughter	Unclear	Low
[48]	High	Low	Unclear	BW180	Low	Low
[49]	High	Low	Unclear	BW100 and BW205	Low	Low
[46]	High	Unclear	Unclear	BW205	Low	Low

^1^Classified as low, unclear and high.

BW60, BW 100, BW180 and BW205 = body weight at 60, 100, 180 and 205 days of age, respectively.

ADG = average daily gain.

LEA = loin eye area.

FT = fat thickness.

### Meta-analysis

Twenty-five publications with 35 studies were included in our MA. The number of publications, studies, trials, and types of outcome measures available for statistical analyses are presented in Table 1.

#### Postnatal body weight of progenies

The variation in the average postnatal calf weight attributable to heterogeneity was high (*I*^2^ = 99.3%; *P*<0.001). The average BW60 was 96.23 kg, and the MD (difference between means) was lower by 1.92 kg (95% CI = -2.616, -1.230; *P*<0.001; n = 3 trials; *I*^2^ = 78.9%) and 1.67 kg (95% CI = -1.924, -1.427; *P*<0.001, n = 2 trials; *I*^2^ = 78.9%) in calves whose mothers consumed CP and ME above their recommended levels, respectively (Table 4). These effects were only significant in the 3TRI (MD = 1.67 kg; 95% CI = -1.924, -1.427; *P*<0.001; n = 2 trials; *I*^2^ = 78.9%), when considered independently of the nutrients.

**Table 4:** Results of meta-analysis for pregnant period, crude protein and metablizable energy consumption by pregnant cows for postnatal weight of calves.

Variable	Average weight (kg)	Studies (trials)	MD^1^ (*P*-value)	IC^2^ 95%	*I*^2^
	*BW60*^*4*^				
Pregnant period					
Second and third trimester	96.23	1 (1)	-5.561 (<0.001)	-8.128, -2.994	0
Third trimester		2 (2)	-1.676 (<0.001)	-1.924, -1.427	0
*Dietary CP level^3^*					
100% *versus* 120%	96.23	3 (3)	-1.923 (<0.001)	-2.616, -1.230	78.9
130% *versus* 180%		1 (1)	-1.786 (<0.001)	-2.135, -1.437	0
*Dietary ME level* *^3^*					
100% *versus* 120%	96.23	2 (2)	-1.676 (<0.001)	-1.924, -1.427	0
	*BW180*^*5*^				
*Dietary CP level^3^*					
120% *versus* 180%	230.99	5 (2)	-0.107 (0.734)	-0.721, 0.508	97.2
60% *versus* 80%		2 (2)	-2.076 (0.413)	-7.046, 2.895	97.7
*Dietary ME level^3^*					
120% *versus* 180%	230.99	6 (3)	-0.262 (0.666)	-1.452, 0.928	98.2
60% *versus* 80%		2 (2)	0.477 (0.326)	-0.476, 1.429	97.5
80% *versus* 120%		1 (1)	-3.909 (<0.001)	-5.361, -2.457	0
	*BW205*^*6*^				
Pregnant period					
First trimester	240.81	1 (1)	1.451 (0.004)	0.455, 2.448	0
Third trimester		8 (4)	-1.150 (0.014)	-2.068, -0.232	97.7
Second and third trimester		2 (2)	-9.305 (<0.001)	-13.136, -5.475	99.9
All pregnancy		2 (2)	3.998 (0.388)	-5.083, 13.078	99.7
*Dietary CP level^3^*					
120% *versus* 180%	240.81	5 (2)	-0.451 (0.433)	-1.578, 0.677	97.2
60% *versus* 80%		4 (2)	-2.216 (0.161)	-5.315, 0.884	98.8
80% *versus* 120%		1 (1)	-1.835 (<0.001)	-2.142, -1.529	0
*Dietary ME level*^3^					
120% *versus* 180%	240.81	6 (3)	-0.499 (0.371)	-1.594, 0.595	95.8
60% *versus* 80%		2 (2)	0.315 (0.821)	-2.412, 3.041	98.9
80% *versus* 120%		3 (1)	-3.122 (<0.001)	-4.299, 1.945	92.6

^1^mean difference in kg.

^2^confidence interval at 95%.

^3^control *versus* treatment.

BW60^4^ = weight at 60 days of age.

BW180^5^ = weight at 180 days of age.

BW205^6^ = weight at 205 days of age.

ME = metabolizable energy.

CP = crude protein.

*I*^2^ = heterogeneity between studies.

No significant results were found for BW100. The average BW180 (n = 31 trials) was 230.9 kg, and a tendency (*P* = 0.055) for weight reduction in calves was observed when their mothers consumed up to 20% less ME than their recommended levels (MD = 0.619 kg; 95% CI = -1.253, 0.014; n = 2 trials; *I*^2^ = 81.2%).

The average BW205 was 240.81 kg (n = 38 trials). No significant differences were found for the CP levels; however, calves weighed less (MD = 3.122 kg; 95% CI = -4.299, -1.945; *P*<0.001; n = 3 trials; *I*^*2*^ = 92.6%) when their mothers consumed up to 120% of the recommended ME levels. This effect was only significant in studies evaluating cows during 2TRI and 3TRI.

#### Average daily gain, age, and slaughter weight of progenies

Animals in all the studies included were maintained in a feedlot during the finishing period. There was no significant effect on the variables tested for slaughter weight (Table 5), with an average animal weight of 566.37 kg (n = 38 trials). However, steers from mothers evaluated at 3TRI were slaughtered 5.5 days before those from other cows (95% CI = -9.918, -1086; *P* = 0.015; n = 3 trials, *I*^*2*^ = 98.5%). The average slaughter age was 452.43 days (n = 8 trials).

**Table 5:** Results of meta-analysis for pregnant period, crude protein and metabolizable energy consumption by pregnant cows for average daily gain, age and slaughter weight.

Variable	Studies (trials)		MD^1^ (*P*-value)	IC^2^ 95%	*I*^2^
*Age at slaughter (days)*					
*Pregnant period*					
First trimester	1 (1)	0.000 (1.000)		-1.240, 1.240	0
Third trimester	3 (1)	-5.502 (0.015)		-9.918, -1.086	98.5
First and second trimester	1 (1)	0.000 (1.000)		-0.462, 0.462	0
All pregnancy	1 (1)	-1.728 (<0.001)		-2.054, -1.401	0
*CP^3^*					
120% *versus* 160%	3 (1)	-5.502 (0.015)		-9.918, -1.086	98.5
*ME^3^*					
120% *versus* 180%	3 (1)	-5.502 (0.015)		-9.918, -1.086	98.5
*Weight at slaughter (kg)*					
*Period of pregnancy*					
First trimester	1 (1)	2.954 (0.002)		1.048, 4.859	0
Third trimester	3 (1)	0.809 (0.461)		-1.339, 2.956	96.4
First and second trimester	1 (1)	0.826 (0.609)		-2.338, 3.991	96.2
All pregnancy	1 (1)	-2.392 (<0.001)		-2.758, -2.026	0
*CP^3^*					
120% *versus* 180%	5 (3)	0.331 (0.753)		-1.727, 2.389	98.4
*ME^3^*					
60% *versus* 80%	1 (1)	2.461 (<0.001)		1.379, 3.542	0
80% *versus* 120%	2 (2)	0.219 (0.935)		-5.019, 5.458	95.7
*ADG*^*4*^ *(kg/day)*					
*Period of pregnancy*					
Fist trimester	1 (1)		1.246 (0.077)	-0.136, 2.627	0
Third trimester	3 (1)		1.366 (0.157)	-0.528, 3.259	97.5
First and second trimester	1 (1)		1.657 (0.072)	-0.148, 3.462	89.9
*CP^3^*					
60% *versus* 120%	1 (1)		1.246 (0.077)	-0.136, 2.627	0
100% *versus* 130%	2 (2)		2.378 (<0.001)	1.589, 3.166	0
130% *versus* 180%	2 (2)		2.274 (<0.001)	1.753, 2.796	0
180% *versus* 200%	1 (1)		0.000 (1.000)	-0.800, 0.800	0
*ME^3^*					
60% *versus* 80%	1 (1)		2.535 (0.157)	1.439, 3.631	0
80% *versus* 180%	2(2)		1.962 (1.000)	0.706, 3.218	51.3
100% *versus* 130%	1 (1)		1.246 (0.077)	-0.136, 2.627	0
120% *versus* 180%	3(2)		1.451 (<0.001)	-0.528, 3.259	97.5
130% *versus* 200%	1 (1)		2.535 (<0.001)	1.439, 3.631	0

^1^mean difference in kg.

^2^confidence interval.

^3^control *versus* treatment.

^4^Average daily gain.

ME = metabolizable energy.

CP = crude protein.

*I*^2^ = heterogeneity between studies.

The ADG in finishing period, when all the studies were included, averaged at 1.692 kg/day (n = 9 trials). The steers of cows from the treated group, i.e., cows consuming 110% to 162% CP, had an ADG 1.476 kg/day higher than that of steers from the control group, i.e., steers of cows consuming 104% to 130% CP (95% CI = 0.129, 2.822; *P* = 0.032; n = 4 trials; *I*^*2*^ = 96.1%). Steers of cows that consumed up to 180% of the recommended [19] ME requirements during pregnancy had 1.451 kg/day higher weight gain than that of cows consuming ME below the recommended levels. There was no significant effect of the pregnancy period for this variable.

#### Loin eye area at slaughter of progenies

The 10 trials evaluating LEA showed an average of 83.18 cm^2^, and there were no significant differences based on the CP and ME consumption, neither for pregnancy period for this variable.

#### Progeny marbling at slaughter

The average marbling was 534.90 points (n = 8 trials). The marbling was 1.689 points higher in calves of mothers evaluated in the 3TRI (95% CI = 0.750, 2.629; *P*<0.001; n = 4 trials; *I*^2^ = 91.7%) (Table 6). The marbling score was 1.689 points higher in steers of cows consuming up to 162% and 120% of the recommended [19] CP and ME levels, respectively (95% CI = 0.750, 2.629; *P*<0.001; n = 4 trials; *I*^2^ = 91.7%), than that for other levels of consumption.

**Table 6:** Meta-analysis results for gestation period, crude protein and metabolizable energy consumption by pregnant cows for loin eye area, marbling and fat thickness at the slaughter of their progenies.

Variable	Studies (trials)	MD^1^ (*P*-value)	IC^2^ 95%	*I*^2^
*LEA (cm*^2^*)*				
Period of pregnancy				
First trimester	1 (1)	0.930 (0.168)	-0.391, 2.251	-
Second trimester	2 (2)	-2.729 (<0.001)	-3.351, -2.108	0
Third trimester	4 (2)	-1.593 (0.084)	-3.399, 0.212	97.8
All pregnancy	1 (1)	-2.406 (<0.001)	-2.772, -2.039	-
*CP^3^*				
120% *versus* 180%	1 (1)	-2.719 (0.002)	-4.447, -0.991	0
50% *versus* 80%	1 (1)	-2.731 (<0.001)	-3.397, -2.065	0
*ME^3^*				
120% *versus* 180%	1 (1)	-2.719 (0.002)	-4.447, -0.991	0
*Marbling (marbling score)*				
*Period of pregnancy*				
First trimester	1 (1)	2.157 (<0.001)	1.033, 3.282	0
Second trimester	1 (1)	-2.716 (0.002)	-4.443, -0.989	0
Third trimester	4 (2)	1.689 (<0.001)	0.750, 2.629	91.7
*CP^3^*				
120% *versus* 180%	4 (2)	1.689 (<0.001)	0.750, 2.629	91.7
100% *versus* 130%	2 (2)	-0.006 (0.998)	-4.731, 4.718	98.1
*ME^3^*				
120% *versus* 180%	4 (2)	1.689 (<0.001)	0.750, 2.629	91.7
100% *versus* 120%	5 (3)	1.170 (0.030)	0.114, 2.226	93.3
*Production system*				
Extensive	2 (2)	-0.038 (0.987)	-4.703, 4.627	98.0
Intensive	2 (2)	2.392 (<0.001)	2.088, 2.696	0
*FT (cm*^2^*)*				
*Period of pregnancy*				
First trimester	1 (1)	0.000 (1.000)	-0.877, 0.877	0
Second trimester	1 (1)	2.869 (0.002)	1.090, 4.648	0
Third trimester	4 (2)	2.359 (<0.001)	2.095, 2.623	0
*CP^3^*				
120% *versus* 180%	4 (2)	2.359 (<0.001)	2.095, 2.623	0
100% *versus* 130%	2 (2)	2.407 (<0.001)	1.910, 2.904	0
*ME^3^*				
120% *versus* 180%	4 (2)	2.359 (<0.001)	2.095, 2.623	0
100% *versus* 120%	5 (3)	2.370 (<0.001)	2.109, 2.631	0
*Production system*				
Extensive	2 (2)	2.318 (<0.001)	1.782, 2.855	0
Intensive	2 (2)	2.372 (<0.001)	2.069, 2.675	0

^1^mean difference in kg.

^2^confidence interval.

^3^control *versus* treatment.

*I*^2^ = heterogeneity between studies.

CP = crude protein.

ME = metabolizable energy.

LEA = loin eye area.

FT = fat thickness.

#### Fat thickness of progenies at slaughter

The average FT was 1.51 cm (n = 8 trials). The FT was higher in steers whose mothers consumed up to 130% and 123% of the recommended [19] CP and ME levels (95% CI = 2.095, 2.623; *P* <0.001; n = 4 trials; *I*^2^ = 0%), during the 3TRI (Table 6).

### Publication bias

**Fig 1:**
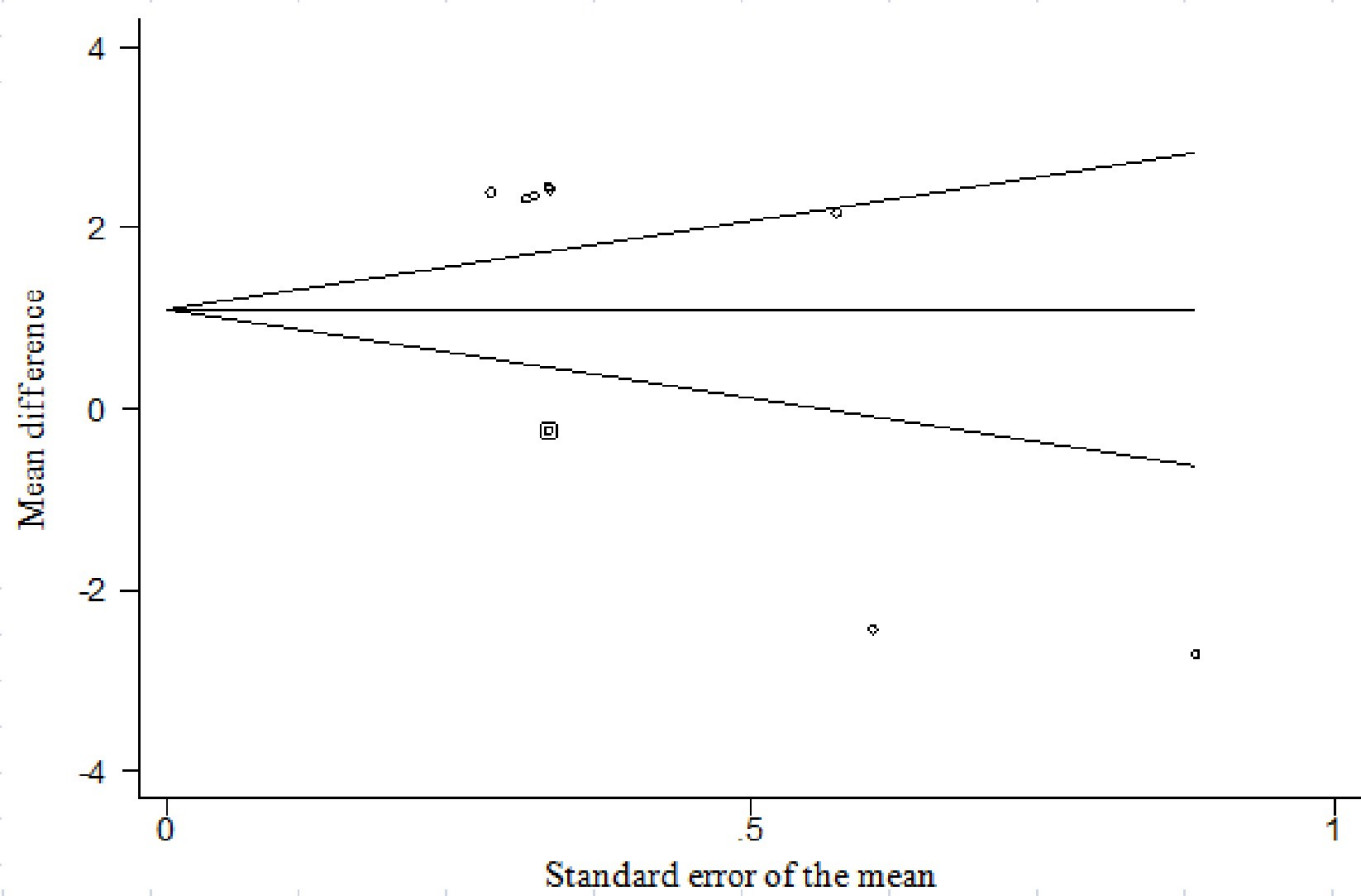
Funnel plot of Duval and Tweedie’s ‘trim-and-fill’ linear random effect model measuring standard for marbling.

The studies included in the current MA showed high heterogeneity, and therefore, the results should be interpreted with caution. The Begg test was only significant for marbling (*P* = 0.048), and the "trim and fill" test indicated that the inclusion of nine trials would be necessary to remove this observed bias (Fig 1).

### Meta-regression analysis

In general, meta-regression should not be considered when there are fewer than 10 studies in the MA [25]. Therefore, this analysis was not performed for the following outcomes: BW60 (n = 3 studies), BW100 (n = 5 studies), age and weight at slaughter (n = 6 studies), ADG at slaughter (n = 5 studies), LEA at slaughter (n = 8 studies), marbling (n = 6 studies), and FT at slaughter (n = 6 studies). For the other variables, i.e., BW180 and BW205, the results were not significant.

### Cumulative meta-analysis and sensitivity analysis

No significant evidence was found in the cumulative meta-analysis. In the sensitivity analysis, the removal of some studies changed the MD of the outcomes BW60, BW100, BW180, BW205, and LEA and marbling at slaughter (Table 7).

**Table 7:** Changes in mean difference of variables weight at 60, 100, 180 and 205 days of age, loin eye area and marbling at slaughter with the removal of studies.

Variable	MD^1^	Amplitude in MD change	Influential study
BW60	-1.923	-3.451	[30]
		-3.351	[40]
BW100	-0.573	-0.400	[6]
		-1.363	[49]
BW180	-0.163	-0.382	[28]
		-0.219	[39]
		-0.277	[31]
		-0.046	[31]
		0.035	[31]
		-0.017	[41]
BW205	-2.983	-0.693	[36]
LEA^2^	-1.654	-2.234	[45]
Marbling^3^	1.299	1.756	[34]
		1.946	[8]
		0.452	[43]

^1^in kg.

^2^in cm^2^.

^3^in marbling score of USDA (100 = Practically devoid; 200 = Traces; 300 = Slight; 400 = Small).

MD = mean difference.

BW60, BW 100, BW180 and BW205 = body weight at 60, 100, 180 and 205 days of age, respectively.

LEA = loin eye area.

## Discussion

If a timeline of results were drawn, it would become evident that the progeny of cows fed with ME and CP levels above their required levels, in 2TRI and 3TRI, weighed significantly less after 60 and 205 days of age. However, their weight gain was higher during the finishing phase, they were slaughtered earlier, and they produced carcasses with higher marbling and fat thickness. The better fat deposition in the carcass may have been due to the high energy and protein intake during pregnancy, favoring hyperplasia of fat cells over the muscle cells [4]. This was confirmed by a reduction in body weight by 60 and 205 days of age. Thus, a negative effect of a high ME and CP intake by cows can be expected on the LEA of their progeny, as this variable represents the percentage of carcass muscles. However, we did not observe any significant effect on this variable, possibly due to the low number of studies included. As noted previously [5], the transfer of nutrients from mother to fetus is particularly important during the late stages of pregnancy, for development of carcass characteristics.

### Postnatal body weight

The intake of CP by pregnant cows influenced only the BW60 when this consumption was evaluated during the 3TRI (Table 4). The BW60 was reduced when the CP intake of pregnant cows was higher than recommended levels [19]. Several reports have shown that the progeny of cows that consume CP above their requirements, during the 2TRI and 3TRI, have lower insulin sensitivity, which directly influences the utilization of glucose by cells, and negatively affects body development [6, 7]. Similarly, as the CP intake of pregnant cows increases, there is a reduction in the circulating IGF-I levels, which is the main hormone responsible for nutrient partitioning between mother and fetus, and a consequent reduction in calf weight [50].

The energy intake of beef cows above the recommended [19] levels during the 2TRI and 3TRI restricted progeny growth up to 205 days (Table 4) of age, and at 180 days, their growth showed a tendency to further decrease. The BW205 decreased with the increase in the ME consumption of pregnant cows, possibly due to the impairment of secondary myogenesis during the 2TRI [51]. If the difference in the effect of maternal nutrition were only in the 3TRI, calves would have hypertrophied muscle cells after birth, since during this phase only cell hypertrophy occurs, as in postnatal life [2].

These findings indicate the crucial role of maternal nutrition during pregnancy, in regulating the differentiation of mesenchymal stem cells in the fetal skeletal muscles into myocytes, adipocytes and fibroblasts, as it is dependent on maternal signals which further depend on the cow’s metabolic status [1]. The high energy levels may have caused a low-grade inflammation in the embryo, resulting in epigenetic changes in the mesenchymal stem cells, which attenuate myogenesis and promote adipogenesis. Since muscle tissue makes up 40% to 50% of the body mass, these results can affect the postnatal weight [2].

This hypothesis can be confirmed by the increase in marbling and FT (Table 6), when cows consumed ME and CP above their recommended levels. This change usually occurs during the embryonic phase, up to the second month of pregnancy [9]. However, our results were significant for 2TRI and 3TRI, possibly due to the low number of publications reporting this data for the first trimester of pregnancy (1TRI). In outher study, [4] observed that cows evaluated during the 1TRI (47 days after conception) and consuming 1.5 times more than the maintenance energy level, produced fetuses with higher fat cell numbers, than the cows fed according to their requirements [19]. The high energy level promoted epigenetic modifications in the fetus, increasing the expression of adipogenesis-linked genes, Zfp423, EBPα and PPARγ [4].

Furthermore, several studies have reported an increase in the free fatty acid levels in the plasma and tissues of pregnant dairy cows [52] from overnutrition, which are the major contributors to fetal insulin resistance [53]. Ewes fed 150% of the recommended [54] energy requirements, for 60 days from conception to calving, produced lambs that were more sensitive to insulin, compared to ewes fed according to their requirements [55]. While insulin resistance in cows can increase fetal glucose availability, it can impair maternal-fetal nutrient transfer, as insulin stimulates synthesis of muscle proteins, and inhibits their degradation. Thus, insulin resistance increases the net rate of proteolysis in the body [6, 56].

Insulin resistance probably compromises the placental supply of nutrients and oxygen during late gestation, causing a reduction in fetal body weight until birth [1]. In pigs and rats with low birth weight due to flaws in muscle development, muscle growth does not recover during the postnatal period, even when fed properly [57]. In pigs, the progeny of sows that consumed more than their energetic requirement, between 0 to 50 days of gestation, showed slower suckling growth rates, compared to piglets born of malnourished sows [58]. The insults caused to the fetus due to failures in maternal nutrition have a long-term effect on their growth and performance [2].

### Average daily gain, age, and weight at slaughter

Intake of CP and ME at higher than the required levels by cows affected the age at slaughter of steers, reducing it by approximately six days, which may be of interest to intensive finishing systems. The ADG during the finishing phase was higher in steers of cows with access to greater dietary nutrients (higher ME and CP content), which can be explained by the accumulation of fat in adipocytes, which were differentiated in greater numbers, at the expense of muscle cells, during the embryonic/fetal period.

Our results are consistent with others already reported [41], who found that the steers of cows supplemented during the later stages of pregnancy had higher ADG in the feedlot period than those of unsupplemented cows. Other authors [59] observed that heifers born of cows fed with CP-rich diet during late gestation tended to be heavier during the period from weaning till breeding. Conversely, several authors found no differences for ADG [6, 7, 40] and slaughter weight [6].

Our results could have been supported by the LEA data, an indicator of carcass muscle development [60]. However, sufficient publications were not available to be able to detect differences.

### Loin eye area at slaughter

In this SR-MA, the nutritional aspects of the 2TRI had a significant impact on LEA (Table 6). Thus, this phase of pregnancy may be a critical period for the development of the carcass muscle portion [61]. According to them, this period promotes quantitative and qualitative changes in the developmental trajectory, with effects that persist throughout life. However, the low number of publications and comparisons available in our database may have prevented the detection of the effect of CP and ME consumption by cows on the progeny LEA. Cows fed with 129% of the recommended [19] CP levels at the end of gestation had no effects on the steers’ LEA, which is in accordance with our results [7]. Furthermore, another study [34] did not observe any effects of feeding pregnant cows with diets providing energy at 100% or 80% of the recommended [19] levels during the 2TRI. However, they reported a tendency for lower LEA in steers of cows maintained at a positive energy status during the 2TRI, compared to those that consumed 80% of their energy requirements [19].

### Marbling and FT at slaughter

There was an increase in marbling and FT at slaughter when cows consumed CP and ME at levels above the recommendations [19] during the 3TRI (Table 6). These results may be related to adipose tissue development during the fetal phase at the expense of the muscle tissue, as previously described. Moreover, these results are consistent with those obtained for BW60 and BW205 (Table 4).

Increased fetal muscle adipogenesis leads to an increase in the number of intramuscular adipocytes, which accumulate fat during postnatal growth, and produce marbling [9]. Therefore, the effectiveness of nutritional management in altering marbling is more evident in the fetal and neonatal phases than at an older age. After 250 days of age, energy consumption becomes less effective in increasing the number of intramuscular adipocytes, due to multipotent cell depletion; however, adipocyte size may increase, resulting in increased marbling during the fattening phase [2]. All animals evaluated in this MA were in the feedlot, which is characterized by high concentrate intake that contributes to glucose uptake by adipocytes [41], as it is the primary substrate used by bovine intramuscular adipocytes [62]. An inflammatory process in the progeny of cows with access to a high nutritional diet produces intramuscular lipogenic responses in steers and contributes to the improvement of fat content in beef [8]. Similarly, overnutrition in ewes during late pregnancy increases the expression of genes associated with adipogenesis and lipogenesis in the perirenal adipose tissue of fetal lambs [63]. Fetal growth progresses rapidly during the last trimester of pregnancy, during which nutritional levels may alter the location of nutrient deposition in tissues [41, 64].

Progenies of pregnant cows fed with 129% of the recommended [19] CP levels during the later stages of gestation had increased 12th rib fat thickness at slaughter, compared to those of cows fed with 100% of the recommended CP levels [7]. These findings corroborate the results of this study, which showed that a higher CP intake in the prepartum diet increases the carcass adiposity (Table 6). A possible explanation for this is that the high-protein contents in the diet of pregnant cows reduces their progenies’ potential for carcass muscle deposition, which energetically provides for greater intramuscular and subcutaneous fat deposition [7].

Several studies on fetal programming in farm animals have shown that maternal malnutrition during pregnancy, up to 60% of the recommended [19] energy and CP levels, reduces weight gain and quality of the progeny’s carcass [8, 12]. Our SR-MA demonstrated that CP and ME up to 180% of the recommended levels are detrimental to calf development during the rearing phase, but advantageous during the finishing phase. It is thus possible to employ nutritional strategies during the prenatal phase to manipulate the production and quality of progeny tissues of commercial interest [2, 9]. This can help to improve the beef quality, while still considering the economic aspects.

## Conclusions

Common sense considers that the greater the amount of energy and, within that, protein, the animals are supplied, the better the productive results. However, our study demonstrate that this advantage does not occur in all productive aspects of beef cattle. In summary, our results suggest that CP and ME consumption above the levels required by pregnant cows, in 2TRI and 3TRI, reduces the body weight of calves at 60 and 205 days of age. At this stage, low body weight is undesirable as it may burden the finishing phase. Conversely, excess intake of CP and ME by cows in 3TRI can also produce positive results, such as reduction in slaughter age of their progenies, increase in ADG, marbling and fat thickness. As our results encompass only the biological dimension, economically viable tools to incorporate these nutritional aspects in the beef production chain need to be identified.

## Supporting information

S1 Fig Number of publications included and excluded in each level. Adapted from [65].
(TIF)

S1 Table Synthesis of the methodological robustness evaluation of 25 publications (35 studies) included in this meta-analysis. (DOCX)

S2 Table Relevant publications and excluded the final database meta-analysis [66–73]. (DOCX)

S1 File PRISMA 2009 checklist. (DOC)

S1 Data (XLSX)
