# Supplementary figures and images for: Pregnant beef cow’s nutrition and its effects on postnatal weight and carcass quality of their progeny
Source: PLoS One. 2020 Aug 27;15(8):e0237941. doi: 10.1371/journal.pone.0237941 (PMC7452729; doi:10.1371/journal.pone.0237941)

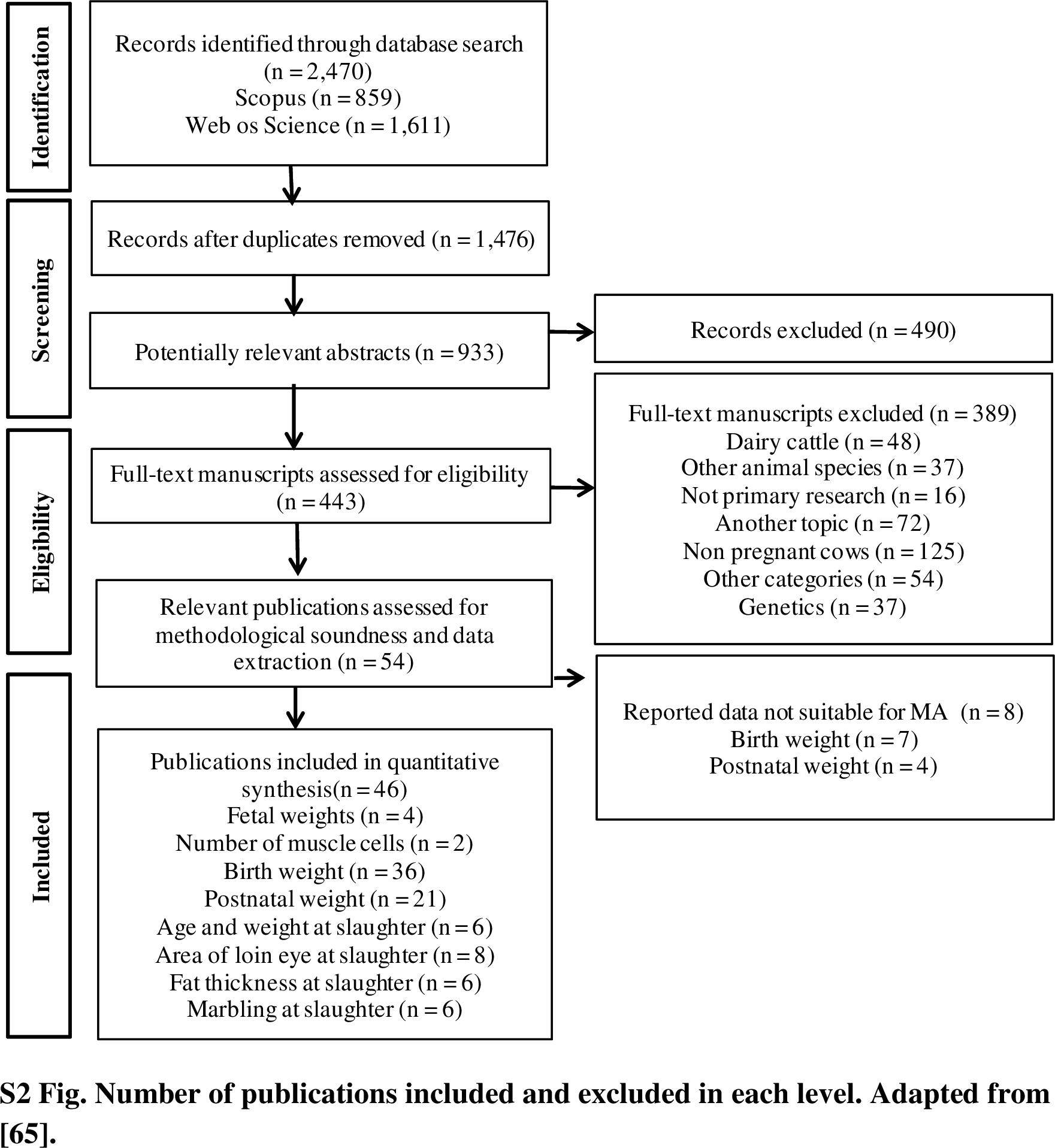

Supplement: S1 Fig — Adapted from [65]. (TIF) [file pone.0237941.s001.tif]
