# Supplementary material for: Pregnant beef cow’s nutrition and its effects on postnatal weight and carcass quality of their progeny
Source: PLoS One. 2020 Aug 27;15(8):e0237941. doi: 10.1371/journal.pone.0237941 (PMC7452729; doi:10.1371/journal.pone.0237941)
Supplement: S1 Table — (DOCX) [file pone.0237941.s002.docx]

**S1 Table. Synthesis of the methodological robustness evaluation of 25 publications (35 studies) included in this meta-analysis.**

| Variable | Evaluation | Weight 60 days | Weight 100 days | Weight 180 days | Weight 205 days | Age and weight at slaughter | Average daily gain | Loin eye area | Marbling at slaughter | Fat thickness |
| --- | --- | --- | --- | --- | --- | --- | --- | --- | --- | --- |
| Was the sample size justified? | Yes | 0 (0) | 0 (0) | 0 (0) | 0 (0) | 0 (0) | 0 (0) | 0 (0) | 0 (0) | 0 (0) |
|  | No | 3 (3) | 5 (5) | 7 (10) | 13 (20) | 6 (6) | 5 (5) | 8 (8) | 6 (6) | 6 (6) |
| How were cows assigned to groups? | Randomization ^1^ | 0 (0) | 0 (0) | 0 (0) | 0 (0) | 0 (0) | 0 (0) | 0 (0) | 0 (0) | 0 (0) |
|  | Random ^2^ | 0 (0) | 0 (0) | 0 (0) | 0 (0) | 0 (0) | 0 (0) | 0 (0) | 0 (0) | 0 (0) |
|  | Systematic ^3^ | 1 (1) | 1 (1) | 1 (1) | 2 (2) | 0 (0) | 0 (0) | 0 (0) | 0 (0) | 0 (0) |
|  | Convenience or purpose ^4^ | 2 (2) | 4 (4) | 6 (9) | 11 (18) | 6 (6) | 5 (5) | 8 (8) | 6 (6) | 6 (6) |
| The intervention protocol was described in sufficient detail to be replicated? | Yes | 3 (3) | 5 (5) | 7 (10) | 13 (20) | 6 (6) | 5 (5) | 8 (8) | 6 (6) | 6 (6) |
|  | No | 0 (0) | 0 (0) | 0 (0) | 0 (0) | 0 (0) | 0 (0) | 0 (0) | 0 (0) | 0 (0) |
|  | Reference document | 0 (0) | 0 (0) | 0 (0) | 0 (0) | 0 (0) | 0 (0) | 0 (0) | 0 (0) | 0 (0) |
| Did the author report that blinding was used to evaluate the outcome? | Yes | 0 (0) | 0 (0) | 0 (0) | 0 (0) | 0 (0) | 0 (0) | 0 (0) | 0 (0) | 0 (0) |
|  | No | 3 (3) | 5 (5) | 7 (10) | 13 (20) | 6 (6) | 5 (5) | 8 (8) | 6 (6) | 6 (6) |
| Based on the study design, was cluster^5^ considered appropriate for the analysis? | Yes | 0 (0) | 0 (0) | 0 (0) | 0 (0) | 0 (0) | 0 (0) | 0 (0) | 0 (0) | 0 (0) |
|  | No | 0 (0) | 0 (0) | 0 (0) | 0 (0) | 0 (0) | 0 (0) | 0 (0) | 0 (0) | 0 (0) |
|  | Not applicable | 3 (3) | 5 (5) | 7 (10) | 13 (20) | 6 (6) | 5 (5) | 8 (8) | 6 (6) | 6 (6) |
| Have confounding factors been identified or controlled? | Yes in the analysis^6^ | 0 (0) | 0 (0) | 0 (0) | 0 (0) | 0 (0) | 0 (0) | 0 (0) | 0 (0) | 0 (0) |
|  | Yes on inclusion/exclusion^7^ | 0 (0) | 0 (0) | 0 (0) | 0 (0) | 0 (0) | 0 (0) | 0 (0) | 0 (0) | 0 (0) |
|  | Yes in correspondence^8^ | 0 (0) | 0 (0) | 0 (0) | 0 (0) | 0 (0) | 0 (0) | 0 (0) | 0 (0) | 0 (0) |
|  | No^9^ | 3 (3) | 5 (5) | 7 (10) | 13 (20) | 6 (6) | 5 (5) | 8 (8) | 6 (6) | 6 (6) |
|  | Not applicable^10^ | 0 (0) | 0 (0) | 0 (0) | 0 (0) | 0 (0) | 0 (0) | 0 (0) | 0 (0) | 0 (0) |
| Has the statistical analysis been adequately described in order to be reproduced? | Yes | 3 (3) | 5 (5) | 7 (10) | 13 (20) | 6 (6) | 5 (5) | 8 (8) | 6 (6) | 6 (6) |
|  | No | 0 (0) | 0 (0) | 0 (0) | 0 (0) | 0 (0) | 0 (0) | 0 (0) | 0 (0) | 0 (0) |
|  | Reference document | 0 (0) | 0 (0) | 0 (0) | 0 (0) | 0 (0) | 0 (0) | 0 (0) | 0 (0) | 0 (0) |
|  | Statistical analysis not performed | 0 (0) | 0 (0) | 0 (0) | 0 (0) | 0 (0) | 0 (0) | 0 (0) | 0 (0) | 0 (0) |

^1^Computer or random number table, a priori, stratified random sample, cluster random sample.

^2^Author(s) report random, but randomization is not described.

^3^“n”samples obtained at x intervals or stratified by certain characteristics.

^4^Author indicated convenience sampling or sampling was not reported in the paper.

^5^Clustering was evaluated when repeated measures were reported.

^6^ Author identified confounders and controlled for them in the analysis.

^7^Confounders were identified and included/excluded a priori.

^8^Confounders were controlled a priori by matching on certain characteristics.

^9^No adjustments were made for confounders/effect modifiers, etc. that were identified by the author.

^10^Confounders were not identified by the author or randomization was used to control for.
