# Supplementary material for: Pregnant beef cow’s nutrition and its effects on postnatal weight and carcass quality of their progeny
Source: PLoS One. 2020 Aug 27;15(8):e0237941. doi: 10.1371/journal.pone.0237941 (PMC7452729; doi:10.1371/journal.pone.0237941)
Supplement: S2 Table — (DOCX) [file pone.0237941.s003.docx]

**S2 Table.** **Relevant publications and excluded the final database meta-analysis [66-73].**

| Reference | Country | Treatment | Reason for exclusion |
| --- | --- | --- | --- |
| [66] | USA | Energy level | Do not show results of interest |
| [67] | USA | Energy level | Do not show results of interest |
| [68] | Hungary | Different forages | Insufficient data on the diet of cows |
| [69] | USA | Protein level | Do not show results of interest |
| [70] | Brazil | Different forages | Insufficient data on the diet of cows |
| [71] | USA | Energy and protein level | Do not show results of interest |
| [72] | Australia | Energy and protein level | There was a change in diet throughout the study |
| [73] | Australia | Protein level | There was a change in diet throughout the study |
